# Peer Review of “Challenges in Implementing a Mobile AI Chatbot Intervention for Depression Among Youth on Psychiatric Waiting Lists: Randomized Controlled Study Termination Report”

**DOI:** 10.2196/82071

**Published:** 2025-09-05

**Authors:** Edel Ennis

**Affiliations:** 1 University of Ulster, School of Psychology, Coleraine, United Kingdom

**Keywords:** randomized controlled trial, AI chatbot, acceptance and commitment therapy, mental health, psychiatry, children, adolescents, Japan

*This is a peer-review report for “Challenges in Implementing a Mobile AI Chatbot Intervention for Depression Among Youth on Psychiatric Waiting Lists: Randomized Controlled Study Termination Report.”*

## Round 1 Review

Thank you for inviting me to review this paper [1]. However, my suggestion would be that this paper should be rejected. I am very cognizant of publication biases, and I am a firm believer that the publication of negative results is very important. I therefore have no problem with the fact that the sample decreased to zero. However, I do believe that more detail is needed in terms of *why* people disengaged. The rationale for the paper is set up as efficacy of the intervention, but the main message of the paper is that the sample declined. I would therefore like more emphasis on qualitative interviews that examined why people disengaged. Follow-up work such as this would make a very interesting paper.

## References

[1] Fujita J, Yano Y, Shinoda S (2025). Challenges in implementing a mobile AI chatbot intervention for depression among youth on psychiatric waiting lists: randomized controlled study termination report. JMIRx Med.

